# Increased functional connectivity between limbic brain areas in healthy individuals with high versus low sensitivity to cold pain: A resting state fMRI study

**DOI:** 10.1371/journal.pone.0267170

**Published:** 2022-04-20

**Authors:** Hadas Grouper, Martin Löffler, Herta Flor, Elon Eisenberg, Dorit Pud

**Affiliations:** 1 Faculty of Social Welfare and Health Sciences, University of Haifa, Haifa, Israel; 2 Medical Faculty Mannheim, Institute of Cognitive and Clinical Neuroscience, Central Institute of Mental Health, Heidelberg University, Heidelberg, Germany; 3 The Rappaport Faculty of Medicine, Technion—Israel Institute of Technology, Haifa, Israel; 4 Institute of Pain Medicine, Haifa, Israel; BG-Universitatsklinikum Bergmannsheil, Ruhr-Universitat Bochum, GERMANY

## Abstract

**Background:**

The representation of variability in sensitivity to pain by differences in neural connectivity patterns and its association with psychological factors needs further investigation. This study assessed differences in resting-state functional connectivity (rsFC) and its association to cognitive-affective aspects of pain in two groups of healthy subjects with low versus high sensitivity to pain (LSP vs. HSP). We hypothesized that HSP will show stronger connectivity in brain regions involved in the affective-motivational processing of pain and that this higher connectivity would be related to negative affective and cognitive evaluations of pain.

**Methods:**

Forty-eight healthy subjects were allocated to two groups according to their tolerability to cold stimulation (cold pressor test, CPT, 1°C). Group LSP (N = 24) reached the cut-off time of 180±0 sec and group HSP tolerated the CPT for an average of 13±4.8 sec. Heat, cold and mechanical evoked pain were measured, as well as pain-catastrophizing (PCS), depression, anxiety and stress (DASS-21). All subjects underwent resting state fMRI. ROI-to-ROI analysis was performed.

**Results:**

In comparison to the LSP, the HSP had stronger interhemispheric connectivity of the amygdala (p = 0.01) and between the amygdala and nucleus accumbens (NAc) (p = 0.01). Amygdala connectivity was associated with higher pain catastrophizing in the HSP only (p<0.01).

**Conclusions:**

These findings suggest that high sensitivity to pain may be reflected by neural circuits involved in affective and motivational aspects of pain. To what extent this connectivity within limbic brain structures relates to higher alertness and more profound withdrawal behavior to aversive events needs to be further investigated.

## Introduction

Studies on brain mechanisms underlying the inter-individual variability in pain perception showed that pre-existing resting-state functional connectivity (rsFC) of pain-related brain regions determines the level of perceived experimental pain [1–4]. Specifically, rsFC of medial-prefrontal and fronto-parietal networks positively associates with heat and pressure pain ratings [5]. Another study found that a pain-free rsFC brain connectome yields a signature pattern that predicts pain sensitivity represented by multimodal pain thresholds [4]. Additionally, [6] showed associations between rsFC of the dorsolateral prefrontal cortex (DLPFC), which modulates the emotional-affective experience of pain and high pain threshold, suggesting that the DLPFC, plays a role in pain modulation, particularly pain tolerance.

Cognitive-affective factors contributing to sensitivity to pain also correspond with differences in cerebral connectivity. For example, pain catastrophizing relates to enhanced connectivity between cognitive-emotional networks in patients with chronic pain [7]. Higher anxiety and pain vigilance among healthy subjects co-vary with lower insular—PAG connectivity [3]. Moreover, decreased connectivity was found between the amygdala and prefrontal regions in response to stress induced by the cold-pressor test (CPT) in healthy individuals [8].

To extend the knowledge on how individual differences in resting-state brain networks are related to individual differences in pain sensitivity and in negative cognitive-affective states, the present study was conducted in two distinct groups of healthy subjects presenting low versus high sensitivity to pain, pre-defined according to their ability to tolerate noxious cold stimulation. In contrast to studies which tested cohorts expressing a continuous range of sensitivities to pain and correlated it with corresponding ranges of imaging results, this dichotomous approach is based on our [9–11], and additional previous reports [12–15], which have consistently observed large inter-individual variability in enduring the CPT. Furthermore, there are pain-tolerant individuals (~15–20% of a sample) who can tolerate the entire CPT and reach the cut-off time, while a similar percentage of individuals withdrew their hand from the cold-water bath within a few seconds. We assumed that the a-priori classification of extreme groups might shed new light on differences between pain-sensitive and pain-tolerant individuals by better distinguishing their differences in rsFC. Therefore, our main aim was to explore the rsFC differences between the two groups across brain regions contributing to the experience of pain. A second aim was to test the relationship between rsFC and negative cognitive-affective states in these groups. Our working hypotheses were a) given the nature of classification of the two groups (pain tolerance, representing motivation to avoid or endure pain), connectivity between affective-motivational areas would be pronounced [16]; b) high sensitivity to pain individuals would demonstrate strong connectivity within affective-motivational brain regions; and c) these brain patterns would positively correlate with negative cognitive-affective states.

## Methods

The study protocol was registered at ClinicalTrials.gov (identifier: NCT03436264), following approval by the ethics committees of the University of Haifa (#165/16) and Rambam Health Care Campus in Haifa, Israel (#0448-17-RMB), and was conducted in accordance with the Declaration of Helsinki.

### Participants

Forty-eight healthy participants were included in the study after meeting the following inclusion criteria: 1) reportedly healthy males and females, over the age of 18, free from chronic pain of any type; 2) no medication use (except for oral contraceptives); 3) able to understand the purpose and instructions of the study and to sign an informed consent. We excluded subjects 1) with a diagnosis of Raynaud’s Syndrome; 2) who were pregnant; 3) with an inability to comply with the study protocol; 4) with metal implants of any kind (including pacemakers); and 5) with claustrophobia.

### Instruments and measurements

The ***Cold Pressor Test (CPT)*** was administered using Heto CBN 8–30 Lab equipment (HETO, Allerod, Denmark). A temperature-controlled water bath with a maximum temperature variance of ± 0.5°C was employed, which was continuously stirred by a pump. Subjects were instructed to immerse their right hand in a water bath at 1°C and to maintain their hand in the cold water for as long as possible. A cut-off time of 180 seconds to hand withdrawal was set for safety reasons. Subjects were instructed to indicate the exact point in time when the cold sensation began to elicit pain. The time until the first perception of pain, measured in seconds (s), was defined as the cold pain threshold. Maximum pain intensity (numerical pain scale, NPS, 0–100) perceived during the immersion was recorded immediately after hand withdrawal. In addition, cold pain tolerance, measured in seconds (s), was defined as the latency of intolerability (spontaneous hand removal).

The ***Thermal Sensory Analyzer*** (TSA-200, Medoc, Ramat Yishai, Israel) was used for assessing heat pain threshold and intensity in two different stimuli. In both tests, a 30x30 mm thermode was attached to the thenar eminence of the dominant hand. Then, (i) a baseline temperature was set to 32°C and increased in a rate of 1°C/s up to a destination of 50°C. Subjects were instructed to press a button when the stimulus began to elicit heat pain (heat pain threshold, °C); (ii) a phasic heat stimulation lasting four seconds at a destination of 46.5°C was delivered (starting from 32°C at an increasing rate of 8°C/sec). Subjects rated the magnitude of perceived pain induced by the stimulus (NPS, 0–100, heat pain intensity).

The ***Pressure Algometer*,** (AlgoMed, Medoc, Ramat Yishai, Israel) was used to assess mechanical pain threshold and tolerance. The device has a rubber hand grip held by a lever and a 1cm2 rubber tip placed on the tested region, between the fingers of the hand. A constant rate of pressure was delivered by the examiner, and the pressure was recorded in Kilopascal (kPa). Subjects were instructed to press a button at the exact point in time when the pressure stimulation began to elicit pain. This point was defined as the pressure pain threshold (kPa-kilopascal). The time point at which the subject could no longer tolerate the pressure pain stimulation was defined as the pressure pain tolerance (kPa).

### Pain catastrophizing

Catastrophizing was assessed by the Pain Catastrophizing Scale (PCS) [17], using the validated Hebrew version [18]. It includes 13 items (using a Likert scale ranging from 0 = “Not at all” to 4 = “very great extent”), representing the three components of pain catastrophizing: rumination (e.g., ‘‘I can’t seem to keep it out of my mind”); magnification (e.g., ‘‘I wonder whether something serious may happen”); and helplessness (e.g., “There is nothing I can do to reduce the intensity of pain”).

### Depression, anxiety and stress

The DASS-21 questionnaire was used for measuring depression, anxiety and stress states. The DASS-Depression Scale focuses on reports of low mood, motivation, and self-esteem, DASS-Anxiety focuses on reports of physiological arousal, perceived panic, and fear, and DASS-Stress on tension and irritability. The DASS-21 questionnaire assesses each scale with 7 Items. Its validity and reliability were tested in patients with chronic pain and in healthy volunteers [19].

### rs-fMRI acquisition

Image acquisition was performed on a 3-Tesla GE Scanner with head, neck, and spine coil (HNS, Milwaukee, WI). We obtained T1-weighted, magnetization-prepared, inversion-prepared, (IR-prep), fast spoiled gradient echo (SPGR) sequence (brain volume, BRAVO) images with the following parameters: repetition time (TR) = 7.172 ms, echo time (TE) = 2.704 ms, flip angle 12°, field of view (FOV): 256 × 256 mm^2^, matrix size: 256 × 256, voxel size: 1.0 × 1.0 × 1.0 mm^3^, and 176 sagittal slices. Blood oxygenation level-dependent (BOLD) contrast whole-brain functional images were acquired using a T2*-weighted gradient-echo Echo Planar Imaging (EPI) sequence (protocol parameters: TR = 2700 ms; TE = 25 ms; matrix size = 80*80; FOV = 200 x 200 mm^2^; flip angle = 60°, with asset factor 2 acceleration). The field of view was chosen to ensure the recording of BOLD contrasts of all cortical regions in all subjects. It further covered upper parts of the brain stem such as the periaqueductal grey (PAG), but not the cerebellum. One-hundred-and-ninety volumes with 40 axial slices (slice thickness = 2.5 mm, with no gap, voxel size = 2.5 mm^3^) were measured in interleaved slice order and positioned along a tilted line to the anterior-posterior commissure (AC-PC orientation). An automated high-order shimming technique was used to maximize magnetic field homogeneity. Before preprocessing, the first 3 volumes of each scanning session were discarded to allow for T1 equilibration effects.

### Experimental design

**Fig 1:**
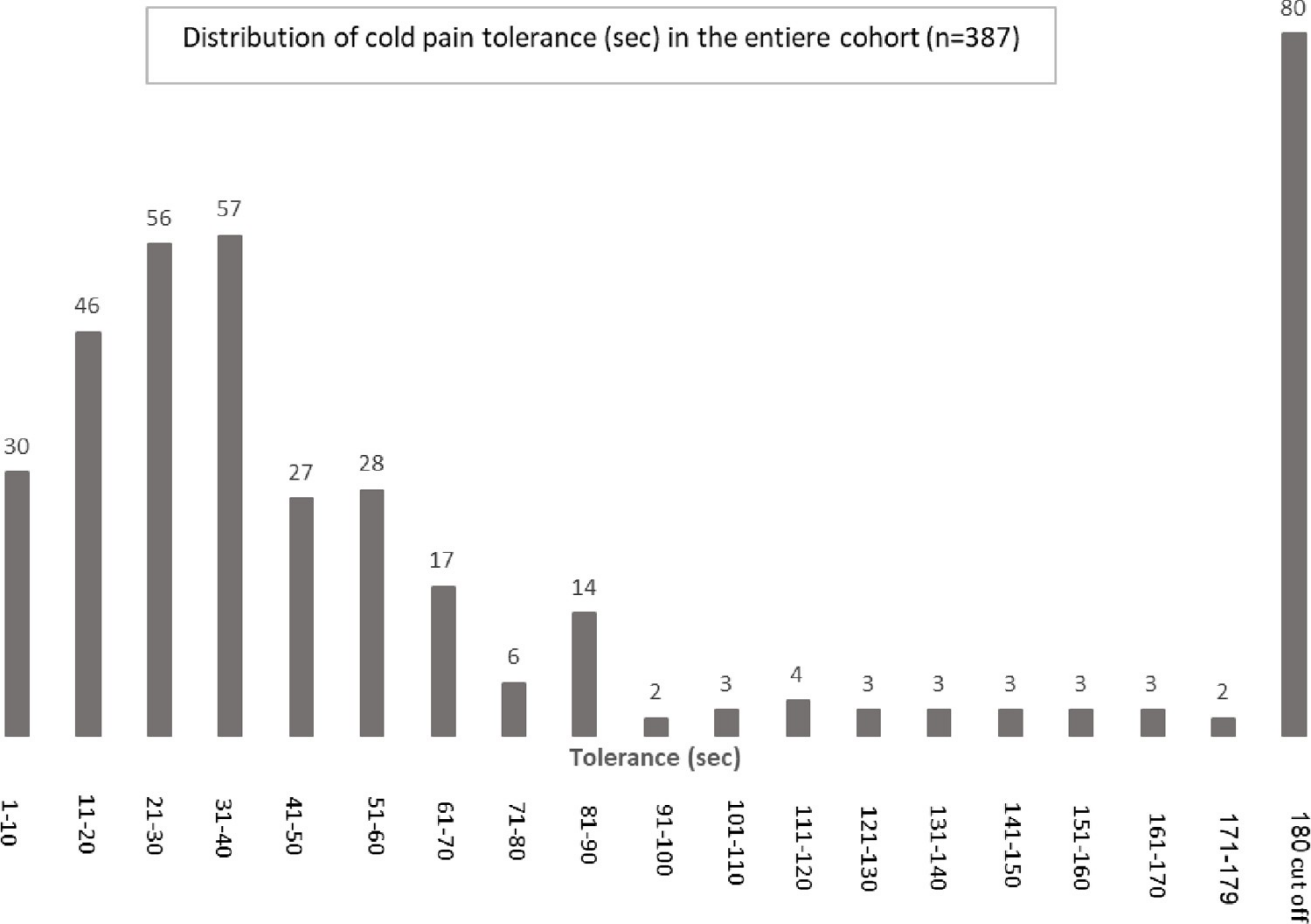
Distribution of cold pain tolerance (sec.) in the entire cohort (n = 387).

The research was conducted between May 2016 –January 2020. Three-hundred-and-eighty-seven participants were recruited through advertisements distributed on a campus bulletin board at the University of Haifa. Responders received a detailed explanation of the study at the beginning of the session. In order to identify two distinct groups of very low and very high sensitivity to pain, subjects’ tolerance to noxious cold stimulation (CPT) was tested. Notably, although sensitivity to pain can be defined by responses to various pain parameters (e.g., latency to pain onset, pain magnitude, pain tolerance, slopes between tolerance and threshold, etc.), in this study we refer specifically to pain tolerance as an indicator of sensitivity to pain. Since cold pain tolerance typically exhibits a hump curve with peaks at the two extremes [20], subjects who reached the cut-off time of 180 s were a priori defined as having low sensitivity to pain (LSP) and a size-matched group of subjects who displayed the shortest tolerance to the CPT were classified as having high sensitivity to pain (HSP). Of the 387 healthy subjects who met the inclusion criteria and underwent the CPT, 156 (97F / 59M) were identified as being LSP (76) or HSP (80) according to their tolerance time (Fig 1). All other participants (n = 231) were considered to have ‘in-between’ pain sensitivity and were released from the study. All 156 participants underwent psychophysical pain tests (differences in these pain tests between the two groups are summarized in S2 Table).

The study was held in two independent experimental sessions, each in a different institution. The first session was held at the Human Experimental Pain Research Laboratory at the University of Haifa and consisted of the pain tests and the PCS questionnaire, and the second session was held at the Rambam Health Care Campus where the DASS questionnaire and the MRI scan were done. The 156 subjects who were classified as HSP or LSP in the first session were offered to participate in the second session. The first 24 LSP subjects who agreed to undergo the fMRI session were included in the study. An attempt was made to identify an age and sex matched HSP group. Markedly, no significant differences were found in the interval (months) between the two sessions in the LSP vs. HSP group (10.8±8.9, 11.8±11.0, respectively). The sample size in the current study was determined according to other common practices in experimental resting state fMRI studies [21–23]. Subsequent to obtaining written consent for the MRI, participants received a detailed explanation on the course and duration of the scans. Subjects were instructed to remain in a supine position with their eyes closed and avoid moving until the end of the study. Immediately after the scan, subjects were asked to complete the DASS questionnaire. Only those who participated in both study sessions were included in the final analysis.

### fMRI data analysis

#### Preprocessing

The resting-state functional MRI data were preprocessed in the functional connectivity toolbox (CONN toolbox, version 18b; http://www.nitrc.org/projects/conn) [24]. The preprocessing included the following steps: a) functional realignment and unwarping; b) functional slice-timing correction; structural segmentation and normalization; c) functional normalization; d) functional outlier detection; and e) functional smoothing with a Gaussian kernel of 8mm full width at half maximum. We further applied a denoising on the first-level analysis using a linear regression followed by a band-pass filter (0.008–0.09 Hz) in order to remove unwanted motions, physiological effects, and other artefactual effects from the BOLD signal before moving on to calculating connectivity measures.

#### rs-fMRI analyses

To assess differences in rsFC across brain regions traditionally associated with sensory-discriminative, affective-motivational, and cognitive-evaluative aspects of pain processing [4,25,26], we performed ROI-to-ROI analyses with predefined ROIs. The seeds used for the analysis were the anterior insula, anterior cingulate cortex, thalamus, primary and secondary somatosensory cortex, (S1, S2), putamen, supplementary motor cortices, primary motor cortex (M1), dorsolateral prefrontal cortex, (DLPFC), ventromedial prefrontal cortex (vmPFC), nucleus accumbens (NAc), hippocampus and the amygdala. This set of particular brain areas has been shown to be involved in pain perception [26] and has also been found to be a part of the brain signature of pain sensitivity, based on the FC acquired during pain-free resting-state fMRI [4]. The co-ordinates of the pain-related cortical regions were chosen according to a meta-analysis of 140 neuroimaging studies that explored the location and extent of pain-related brain activations in response to noxious stimuli in healthy volunteers [27]. Coordinates for subcortical regions related to the affective component of pain (hippocampus, amygdala) were chosen according to Bingel et al [28]. The coordinates for NAc were chosen according to the Brainnetome Atlas [29]. The coordinates were chosen for each region in the right and the left hemisphere and aligned to the MNI152 standard space (http://sprout022.sprout.yale.edu/mni2tal/mni2tal.html) [30], with 10 mm sphere size masked for each seed (the specific regions with their MNI coordinates are provided in S1 Table). An ROI-to-ROI analysis was performed using the CONN software. The connectivity between all ROIs was compared between LSP and HSP groups and the threshold was defined as α < .05 (*p*-FDR seed-level corrected; two sided).

### Statistical analysis

We used independent *t*-tests or Mann Whitney tests (for parametric or non-parametric variables, respectively) to compare the groups in their psychophysical results and the cognitive-affective factors of pain catastrophizing, depression, stress and anxiety. Cohen’s *d* was used to indicate the effect size for the mean difference comparisons between groups. A Bayesian null-hypothesis testing with Jeffreys’s Amazing Statistics Program 0.14.0.0 and zero-centered standard medium-width priors was used to indicate the bayes factor (BF) of the demographic variables (age and gender). To further minimize any bias related to age and gender we included these variables as covariates into our analysis. Two-sample *t*-tests were performed to examine the differences in brain FC between the groups. The statistical results were used to determine brain regions showing significant differences in terms of BOLD signal time series synchronization between seed regions. The group-level results were corrected for multiple comparisons (false discovery rate—FDR) at seed-level (p<0.05) within the CONN toolbox. Seed-level correction is a parametric correction method offered in CONN that corrects for multiple comparisons arising from testing significance of FC between multiple target ROIs. The significance of ROI-to-ROI connection by intensity was determined through the FDR-corrected p < 0.05 with seed-level correction. Pearson correlation coefficients were calculated between the individual FC values of the correlated regions and cognitive-affective pain-related factors in each group separately. All analyses were performed with SPSS for Windows Version 25 statistical package (SPSS, Inc., Chicago, IL). Significance was declared at p < 0.05. In case analyses included multiple comparison, Bonferroni post-hoc corrections were made, and the specific *p* value required is depicted in the result section for each test.

## Results

### Participant characteristics, CPT and questionnaires

Forty-eight subjects were included in the present study, 24 in each group. The LSP group (pain tolerance of 180±0 sec), 11F / 13M, ranged in age from 20 to 44 years old (mean±SD, 26.4±6.0 years). The HSP group (pain tolerance of 13.3±4.8 sec), 15F / 9M, were aged 18–35 years old (mean±SD, 24.5±3.6). Age and gender did not significantly differ between the groups (t-test and chi square, respectively; p = 0.20, for each). The Bayesian null-hypothesis testing showed anecdotal to moderate evidence for the null hypotheses, i.e., confirmed that the age of HSP did not differ from LSP (BF_01_ = 2.644) and anecdotal evidence that gender of HSP did not differ from LSP (BF_01_ = 1.520). Significant differences were found in all pain tests (in addition to cold pain tolerance) between the groups. Namely, the HSP group demonstrated significantly lower cold heat and mechanical pain thresholds, higher heat and cold pain intensity ratings and lower mechanical pain tolerance compared to the LSP group (p ≤ 0.001). In addition, within the HSP group, in which some variations in cold pain tolerance existed, positive correlation between cold pain threshold and cold pain tolerance (i.e., slope) was demonstrated (*r* = 0.50, p = 0.01). Notably, this was analyzed in the HSP group only since all participants in the LSP group had an identical cold pain tolerance time. The HSP group scored significantly higher in the PCS than the LSP group (p = 0.002) on all three subscales (rumination p = 0.01, magnification p = 0.03, and helplessness p = 0.001). Stress, anxiety and depression did not significantly differ between the groups (Mann-Whitney test, p = 0.30 and p = .15, p = 0.63) (Table 1).

**Table 1:** Group comparisons of the study measures.

	LSP (*n* = 24)	HSP (*n* = 24)			
Characteristics	*Mean ±* SD	*Mean ± SD*	*t Test/ Mann-Whitney*	*p*	*Cohen’s d*
***Pain tests***					
Cold pain threshold (sec)	16.9***±***10.6	3.8***±***2.4	5.8	**<0.001**	1.7
Cold pain intensity (NPS, 0–100)	59.0***±***30.5	79***±***20.8	-2.6	**0.01**	0.8
Cold pain tolerance (sec)	180***±***0.0	13.3***±***4.8	167.6	**<0.001**	-
Heat pain threshold (°C)	45.0***±***4.15	40.9***±***2.9	4.0	**<0.001**	1.14
Heat pain intensity (NPS 0–100)	28.2***±***28.8	92.5***±***11.2	-10.1	**<0.001**	2.9
Mechanical pain threshold (KPa)	508.9***±***387.4	225.5***±***73.7	3.51	**0.001**	1.0
Mechanical pain tolerance (KPa)	1412.8***±***483.8	626.9***±***229.4	7.1	**<0.001**	2.0
***Cognitive-affective states***					
PCS-total score	21.9***±***10.9	32.2***±***10.8	-3.2	**0.002**	0.9
PCS-rumination	8.4***±***4.7	11.5***±***3.8	-2.5	**0.01**	0.1
PCS-magnification	3.8***±***2.9	5.7***±***3.1	-2.1	**0.03**	0.6
PCS-helplessness	9.7***±***5.1	14.8***±***4.9	-3.4	**0.001**	1.0
Stress	6.5***±***7.7	7.7***±***7.2	-1.0	0.3	-0.02ǂ
Anxiety	2.1***±***2.4	4.7***±***6.5	-1.4	0.1	-0.04ǂ
Depression	5.6***±***8.3	5.6***±***7.1	-.50	0.6	-0.005ǂ

*Note*, HSP = High Sensitivity to Pain; LSP = Low Sensitivity to Pain; NPS = numerical pain scale; KPa = kilo Pascal; PCS = Pain Catastrophizing Scale; SD = standard deviation; ǂ = η^2^; s = seconds.

### fMRI results

#### ROI-to-ROI

**Fig 2:**
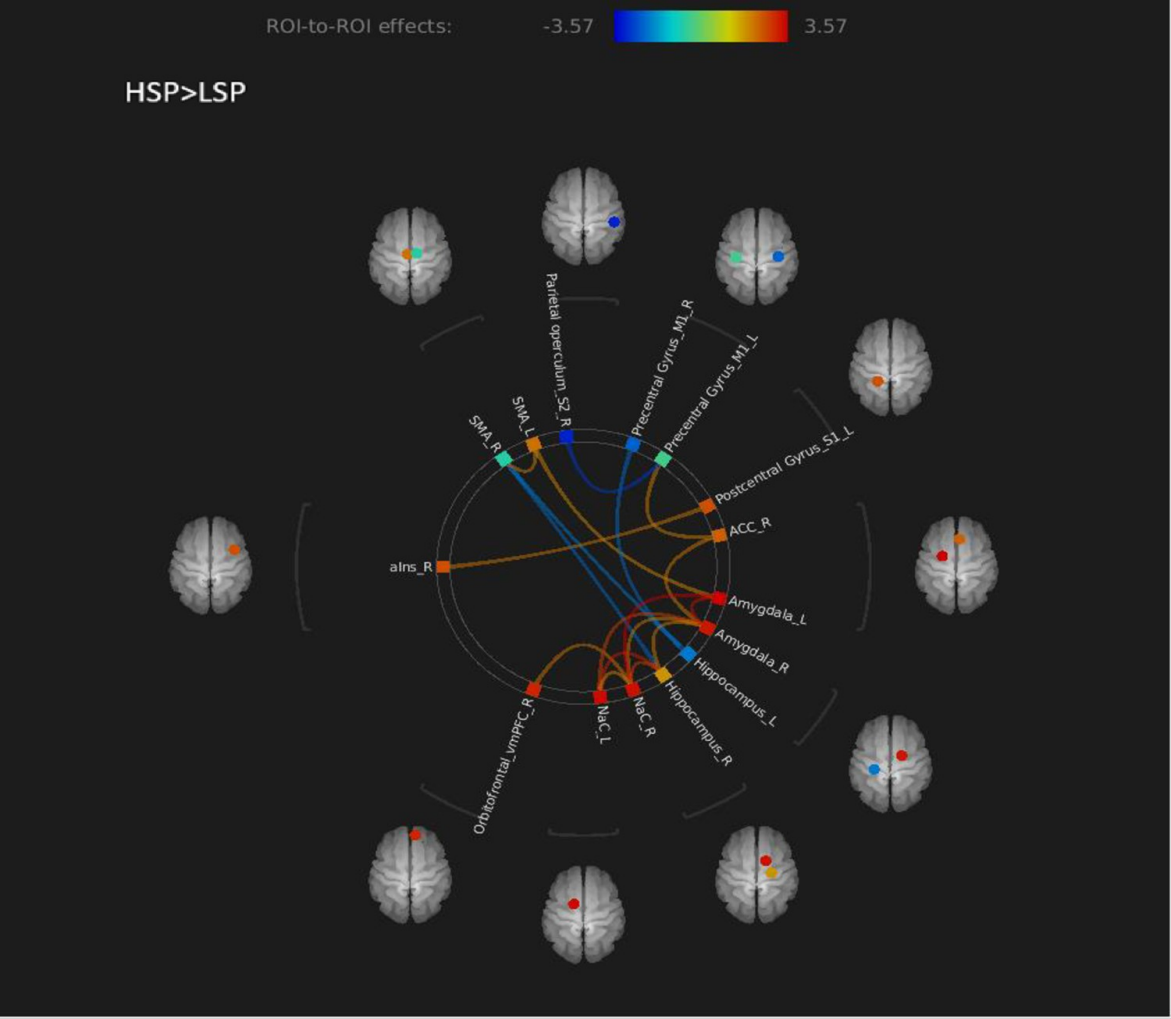
Region of interest (ROI)-to-ROI connectome ring map of the selected ROI seeds in HSP>LSP contrasts. The color links are obtained at a ROI-to-ROI connections height threshold (false discovery rate, FDR) of *P* < 0.05. The color bar indicates the statistical T value. Abbreviations: HSP = high sensitivity to pain; LSP = low sensitivity to pain; NAc = nucleus accumbens; SMA = supplementary motor area; ACC = anterior cingulate cortex; vmPFC = ventromedial prefrontal cortex; aIns = anterior Insula.

**Fig 3:**
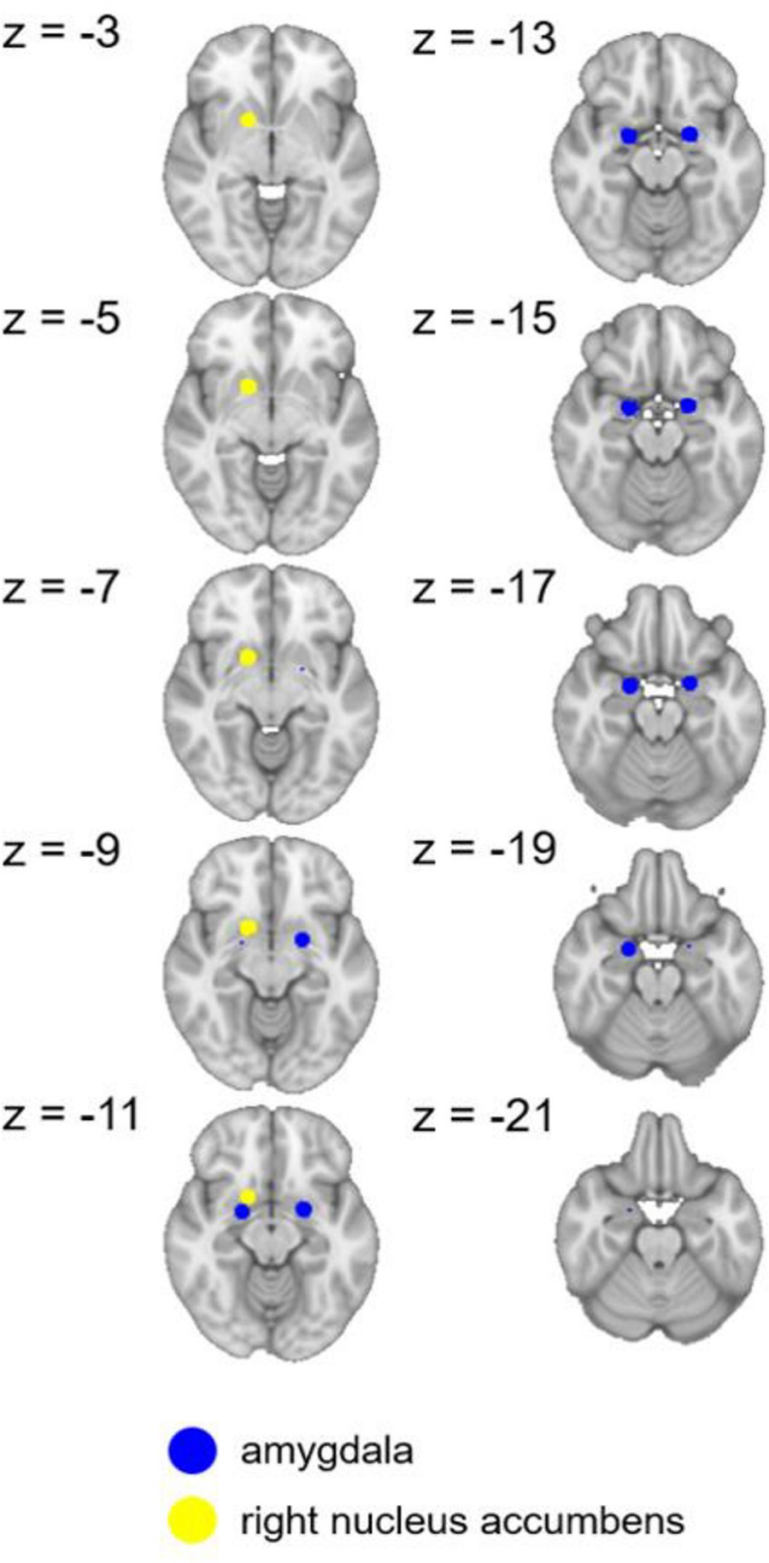
A presentation of the amygdalae and nucleus accumbens ROI’s precise locations in axial slices. These seeds had the most significant functional connectivity in the HSP>LSP contrast. HSP = high sensitivity to pain; LSP = low sensitivity to pain. Coordinates are given in MNI space.

The ROI-to-ROI FC connectome ring (Fig 2) shows that the HSP>LSP contrast had 18 ROI-to-ROI connections. In two of them, statistically significant connectivity differences between the groups were found, based on a false discovery rate (FDR) correction (Table 2). Namely, the HSP group showed significantly stronger BOLD signal time series synchronization between the left and right amygdala and between the left amygdala and the right nucleus accumbens (NAc) than the LSP. Fig 3 shows the precise locations of the amygdalae and NAc. A significant group effect was found after adjusting the connectivity values for age and gender, (for the left and right amygdala connectivity: F (1,47) = 8.8, p = 0.005; for the left amygdala—right NAc connectivity: F (1,47) = 10.3, p = 0.002).

**Table 2:** HSP > LSP contrast ROI connections with threshold ROI to ROI connections by intensity at a 0.05 FDR-P value.

Analysis unit	Statistics (*T*)	*p-*unc	*p-*FDR
Amygdala L- NAc R	3.60	0.0009	**0.01**
Amygdala L- Amygdala R Hippocampus R- NAc L Amygdala R- NAc L Hippocampus R- NAc R	3.40 3.22 2.83 2.80	0.0014 0.002 0.007 0.007	**0.01** 0.06 0.08 0.09
Amygdala L-SMA L SMA R- Hippocampus R SMA R- Hippocampus L SMA R- SMA L Hippocampus R- Amygdala R Precentral Gyrus L- Parietal operculum R Precentral Gyrus L- ACC R Amygdala R- NAc R Amygdala R- ACC R NAc R- vmPFC R NAc R- NAc L Precentral Gyrus R- Hippocampus L Postcentral Gyrus L- aIns R	2.03 -2.30 -2.12 2.07 2.10 -2.87 2.09 2.17 2.16 2.26 2.06 -2.26 2.20	0.04 0.02 0.03 0.04 0.035 0.006 0.042 0.035 0.036 0.02 0.045 0.02 0.03	0.40 0.36 0.36 0.36 0.22 0.15 0.52 0.18 0.18 0.22 0.22 0.56 0.82

*Note*, HSP = high sensitivity to pain; LSP = low sensitivity to pain; NAc = nucleus accumbens; SMA = supplementary motor area; ACC = anterior cingulate cortex; vmPFC = ventromedial prefrontal cortex; aIns = anterior Insula; L = left; R = right; *p-*unc = p-value uncorrected; *p*-FDR = false discovery rate; T = two tailed *t*-test.

#### Within group analysis by gender

Within group analysis by gender found no significant differences in neither the pain measures nor the cognitive affective factors (p > 0.06). Notably, in the LSP group, slightly higher mean brain values of the interhemispheric amygdala connectivity were found in women compared to men (0.4±0.1 versus 0.3±0.1, *z* = -1.9, p = 0.04).

#### Correlations between connectivity patterns and PCS

**Fig 4:**
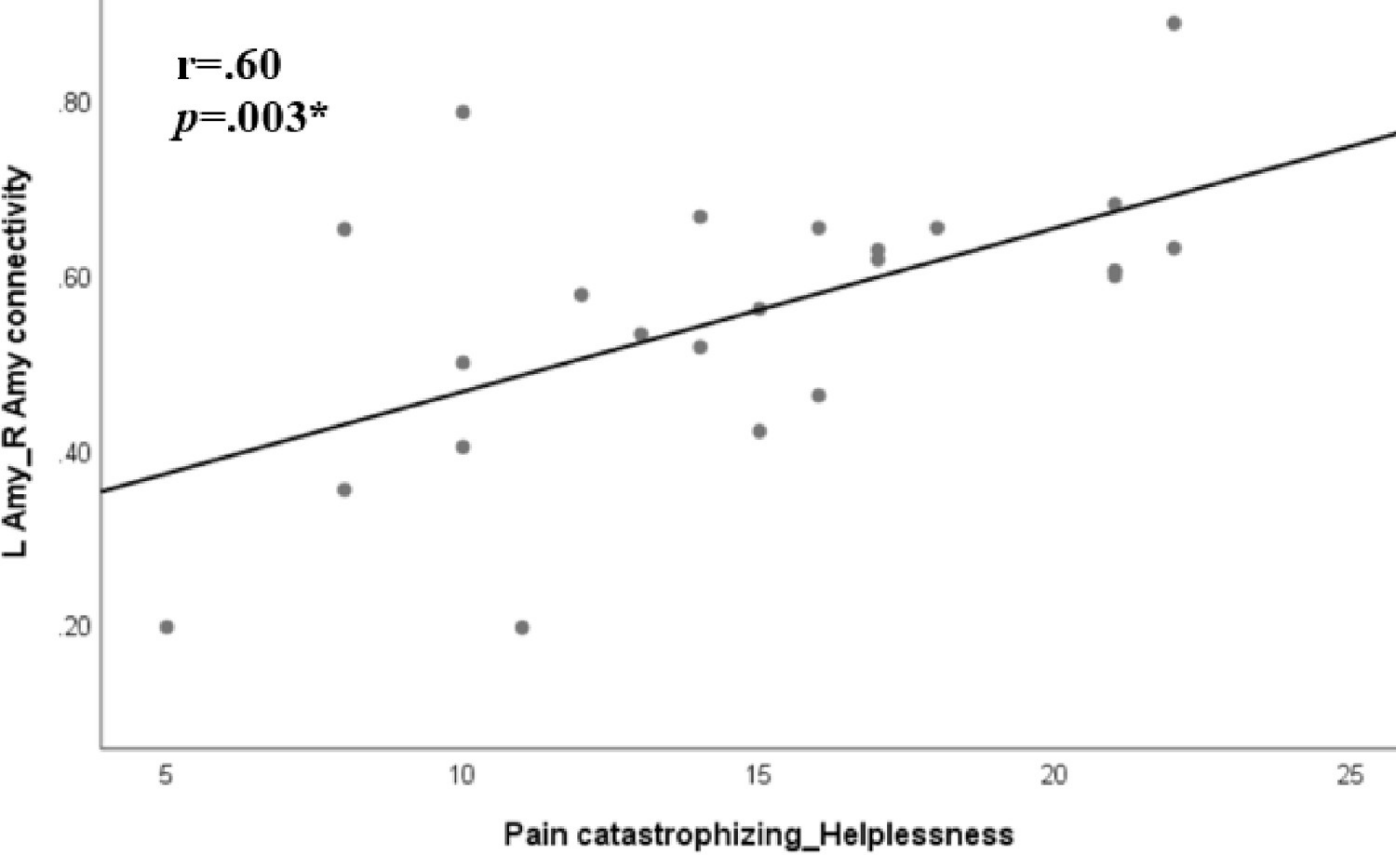
Pearson correlation between pain catastrophizing helplessness subscale and the left amygdala-right amygdala connectivity in the HSP group. *Note*, HSP = high sensitivity to pain; LSP = low sensitivity to pain; Amy = amygdala; L = left; R = right; *Significant at α ≤0.0031 level.

Next, we examined the relationship between pain catastrophizing and the connectivity patterns within each group (Table 3). In the HSP group, the pain catastrophizing helplessness subscale correlated with the amygdala L-R connectivity (*r* = 0.6, p = 0.003) (Fig 4). Additionally, the pain catastrophizing total score reached a trend of significant correlation with the amygdala L-R connectivity (*r* = 0.5, p = 0.009, due to multiple comparisons findings are considered to be significant at p = 0.0031 level). No significant correlations were found in the LSP group. Within groups analyses failed to demonstrate correlations between DASS and Amygdala L-Amygdala R and Amygdala L- NAc R connectivity in the LSP and HSP groups.

**Table 3:** Pearson’s correlations between the connectivity strength of the ROIs and pain catastrophizing in each group.

	PCS-total	Rumination	Magnification	Helplessness	Amygdala L- NAc R
HSP (n = 24)	r (*p*)	r (*p*)	r (*p*)	r (*p*)	r (*p*)
Amygdala L- NAc R	0.09 (0.70)	0.14 (0.50)	-0.11 (0.60)	0.15 (0.50)	-
Amygdala L-Amygdala R	0.52 (0.009)	0.41 (0.04)	0.37 (0.07)	**0.60 (0.003)**	0.50 (0.01)
LSP (n = 24)	r (*p*)	r (*p*)	r (*p*)	r (*p*)	r (*p*)
Amygdala L- NAc R	0.18 (0.40)	0.12 (0.60)	-0.00 (0.90)	0.28 (0.20)	**-**
Amygdala L-Amygdala R	0.11(0.60)	0.05 (0.80)	0.03 (0.90)	0.17 (0.40)	0.50 (0.01)

*Note*, HSP = high sensitivity to pain; LSP = low sensitivity to pain; PCS = Pain Catastrophizing Scale; L = left; R = right. Bold–statistically significant after Bonferroni corrections for multiple comparison, p<0.0031.

## Discussion

The main findings of this study are: (1) differences in rsFC between the groups were found in affective-motivational brain areas; (2) the HSP group demonstrated higher connectivity between the two amygdalae and between the amygdala and NAc than the LSP group; and (3) the connectivity between the amygdalae positively correlated with pain catastrophizing in the HSP group only.

The interaction between the amygdala and the NAc has previously been found to contribute to emotional and motivational aspects of pain [31]. Imaging studies demonstrated activation of limbic areas in various clinical pain states, and the FC of the NAc with prefrontal cortical brain areas has been shown to have a predictive value for the development of chronic pain [32,33]. However, resting state imaging studies in healthy individuals who are either sensitive or insensitive to pain have not yet been conducted. The current study broadens the understanding of this pain-brain relationship by showing that specific limbic brain regions are highly connected in the natural resting state in pain-free individuals demonstrating high pain sensitivity. Looking at the extreme ends of the sensitivity to pain may suggest protective (LSP) or risk (HSP) predisposition factors for the future development of clinical pain. Indeed, patients with neck pain showed higher sensitivity to experimental pain than healthy subjects [34]. Whether this higher sensitivity is a predisposing factor to- or the consequence of- the clinical pain is not yet known. However, based on the higher connectivity between limbic brain areas among healthy HSP individuals that has been demonstrated in our study, one may speculate that people with this pattern of connectivity may tend to be more susceptible for developing clinical pain.

The HSP group showed strong rsFC between the two amygdalae, a limbic brain structure involved in emotions and affective disorders. It has a critical role in emotional processing of pain and in its negative affective experience [35,36]. Healthy volunteers have shown activation of the amygdala in response to nociceptive stimuli of different modalities [37], and the degree of activation was associated with perceived pain intensity [38,39]. Furthermore, resting state studies have shown a higher FC of the amygdala with the insula, midbrain and sensorimotor regions and with the DMN network in patients with different chronic pain disorders [40,41]. Similarly, the HSP group in the current study showed increased connectivity between the amygdalae in a natural resting state position while not being exposed to any noxious stimulus. We suggest that increased neural connectivity between the amygdalae may reflect emotional aspects of existing pain. Also, it may serve as a fingerprint identifying healthy subjects who have high pain sensitivity. The amygdala is further critically involved in fear learning [42], and patients with chronic pain show maladaptive fear learning behaviour [43,44]. High interconnectivity of the amygdalae in HSP subjects might therefore represent a bias towards nociception-induced threat learning processes following a painful event [45], by attaching emotional value to nociceptive inputs [46,47] and, as mentioned earlier, may represent a predisposition for the development of clinical pain. Also, high connectivity within the amygdala may lead to higher processing of danger signals and thus contribute to anticipation/prediction of potentially harmful nociceptive stimulation [48]. Accordingly, in individuals with high sensitivity to pain the amygdala may constantly monitor the environment for such signals and modulate their moment-to-moment alertness level [49]. It is known that the amygdala functions as part of an early alarm system that rapidly detects threat and coordinates defensive behaviors [50] to initiate an immediate affective response [48]. This might also apply to nociceptive input. In contrast, a lack of such connectivity in the LSP group suggests that LSP individuals may have a higher ability to regulate pain and negative emotions as reflected by their ability to tolerate pain. Indeed, a low level of amygdalar function has previously been linked to successful regulation of emotions and pain [51].

The HSP group demonstrated positive connectivity between the amygdala and the NAc. These regions have been identified as part of the brain’s emotion circuitry, which is central to reward and motivated behavior [33], and they influence pain by modulating activity in nociceptive circuits [52]. The amygdala participates in neural circuits underlying behavioural responses to information predictive of aversive events [53] and reward [54]. The NAc encodes signals predictive of the affective value of pain-related information [52], contributes to reward-seeking behavioral responses [55] and reflects motivated behavior to seek relief of pain [56]. The increased connectivity between the NAc and the amygdala in the HSP group may reflect an augmented interaction of the amygdala and the NAc via a direct projection [49]. This excitatory projection of the amygdala to the NAc is essential for eliciting reward-seeking behavioural responses [57]. The predictive value of sensory information is detected and rapidly processed by amygdala neurons. This information is transferred to the NAc, which promotes the decision to initiate a reward-seeking behavioural response. Indeed, the NAc is ideally situated to translate reward-predictive information from the amygdala into reward-seeking motor behaviour [57]. Accordingly, the connectivity between these two regions may function to select actions that optimize reward-seeking in response to sensory information [58]. It can be postulated that in HSP individuals, pain provokes the motivation to escape, terminate, or avoid tissue-damaging processes [59] that provide an aversive memory, thus enabling them to avoid future harm [60,61]. Withdrawing the hand from the CPT was the basis for affiliating our subjects to one of the two groups. Hence, the mesolimbic circuitry of HSP individuals may be highly engaged in predictions of future outcomes of aversive events and may be highly activated in response to noxious stimuli. This can be best understood in the context of HSP individuals’ motivation to seek behavioral goals resulting in the escape or avoidance of pain and the seeking of relief, as well as learning how to predict dangerous or rewarding situations in the future [62]. It has previously been shown that a high predisposition for activation in reward-related circuitry is predictive of the development of pain symptoms [63,64]. The LSP group in our study did not show a positive connectivity between the amygdala and the NAc, suggesting that this group might be less prone to seeking reward and pain relief on the basis of a less active amygdala- NAc connectivity pattern. Thus, this group showed high tolerability to pain.

Notably, it is important to mention other regions involved in affective-motivational processes that did not show connectivity patterns that distinguished between our HSP/LSP groups. For connectivity of the amygdala with other regions, a recent study by Egorova et al., [65] observed heterogeneity of individual responses to heat pain stimulation and found that the ‘more pain’ group had increased right amygdala fMRI activation in response to identical pain stimuli as well as increased resting state connectivity of the left-amygdala and striatum (including the caudate, putamen and nucleus accumbens), compared to the ‘less pain’ group. Regarding the NAc, this region is closely connected with various pain-modulating structures and shares functions involving motivational and emotional processing [66], although only the NAc-Amy pathway dissociated the HSP/LSP groups. Another study showed that NAc activity distinguishes chronic pain patients from healthy subjects by demonstrating that these activity differences correlate with changes in functional connectivity between NAc and other limbic forebrain areas [64]. Specifically, chronic pain patients demonstrated higher NAc-mPFC connectivity than healthy subjects. The authors concluded that these connectivity differences may contribute to the NAc activity that distinguishes the two groups [64]. The vmPFC may play a role in representing the affective and motivational qualities of pain that contribute to its strong aversive drive and was found as a critical link between cognitive state and the affective-motivational features of pain [67]. Yet, no significant differences were found in the connectivity of this region between our groups. With regard to affective-emotional aspects of pain, the connectivity of the DLPFC region would be expected to show differences between the groups, as the FC of this region was previously found to modulate the emotional-affective experience of pain [6]. Specifically, Sankarasubramanian et al. [6] found that individuals with high pain tolerance demonstrate greater changes in FC with DLPFC suggesting that DLPFC is involved in regulating pain tolerance. As both the vmPFC and DLPFC connectivity previously correlated with affective-emotional-motivational aspects of pain [68], we would expect that the FC of these regions would differentiate between the groups, which we failed to demonstrate in the current study”. Possible explanations for the lack in demonstrating those aforementioned differences between the groups may be due to the fact that the present study did not include: 1) chronic pain patients but healthy subjects only 2) the full continuum distribution of pain sensitivity but the extreme ends only (as they may represent a risk groups [34]).

Another finding is the correlation between amygdala connectivity and pain catastrophizing helplessness subscale in the HSP group. Exaggerated amygdala connectivity has previously been found to positively associate with PCS total scores and its subscales rumination and helplessness in patients with chronic back pain [7]. The amygdala connectivity correlation with the helplessness subscale may reflect an expectation of uncontrollability over aversive events [69]. Perceived uncontrollability of pain stimuli in healthy subjects has been associated with activation of the amygdala [70]. Our finding extends this amygdala-catastrophizing relationship to connectivity during the resting state in the HSP group, and suggests that the amygdala may modulate the sensitivity to pain through cognitive factors such as perceived uncontrollability of pain, which has been shown to be associated with greater pain-related suffering [71]. Despite the well-established evidence on the relationship between the other affective factors measured in this study (depression, anxiety, stress) and pain sensitivity [72,73], in the setting of the current study we failed to demonstrate such associations; either by the non-significant difference in scoring between groups or by not showing correlations with brain connectivity, as was found with PCS. We believe that further studies using the same grouping approach are needed in order to substantiate these findings.

Some limitations of the present study should be addressed: First, the study setting was based on two extreme groups and therefore it may limit the generalizability of the results to the entire population. However, as already mentioned, in contrast to the natural continuum seen in the clinical setting, cold pain tolerance does not show a continuum, rather it typically exhibits a hump curve with peaks at the two extremes [20]. Hence, the approach of dichotomizing a natural continuum did not allow continuous/linear analysis strategies of all participants. Second, our analysis approach was based on the selection of specific ROIs. We did not include subcortical regions such as the PAG, raphé nuclei or lobule V and crus II of the cerebellum, which have all been recognized as part of a pain-sensitivity predictive network [4]. Brainstem regions are subject to high physiological noise [74] and appropriate correction of these artefacts could not be carried out as physiological recordings were not possible at the MR facility. Further, the current FOV did not cover cerebellar structures. These regions should be investigated in future studies.

Third, the experimental pain and imaging testing were performed in different sessions. This may be a limitation. However, since both rsFC and CPT tolerance yield repeatable results and likely represent traits rather than states [75,76], this time gap should not have influenced the data. Fourth, there were more women than men in the HSP group and vice-versa in the other group, but these differences between the two groups were not statistically significant. Fifth, participants’ baseline skin temperature, which theoretically might have influenced their pain tolerance, was not measured. Nonetheless, all experiments were performed in a controlled room temperature (25°C). Thus, further studies, using a cohort sample are warranted in order to verify our findings and conclusions.

In conclusion, our study found that individual differences in rsFC are relevant to individual differences in pain sensitivity. As such, an individual’s sensitivity to pain may be encoded in meso-limbic brain regions and can be identified from their resting state brain connectivity. This indicates that high sensitivity to pain may be attributed to resting neural brain patterns related to the affective-motivational dimension of pain, and cognitive/emotional factors. Also, these findings demonstrate the potential of using a rsFC to build a ‘neural trait’ for characterizing an individual’s pain-related behavior, which may eventually contribute to personalized clinical pain assessments.

## Supporting information

S1 Table MNI (x,y,z) co-ordinates of seed ROIs (Bingel et al., 2002 [28]; Duerden and Albanese, 2013 [27]; Fan et al., 2016 [29]). (DOCX)

S2 Table Pain parameters in HSP and LSP in the entire study population (N = 156). (DOCX)
