# Supplementary material for: Increased functional connectivity between limbic brain areas in healthy individuals with high versus low sensitivity to cold pain: A resting state fMRI study
Source: PLoS One. 2022 Apr 20;17(4):e0267170. doi: 10.1371/journal.pone.0267170 (PMC9020745; doi:10.1371/journal.pone.0267170)
Supplement: S1 Table — (DOCX) [file pone.0267170.s001.docx]

**Table S1.** **MNI (x,y,z) co-ordinates of seed ROIs (Bingel et al., 2002; Duerden and Albanese, 2013; Fan et al., 2016).**

| **Region of interest MNI coordinates** | | | |
| --- | --- | --- | --- |
|  | ***X*** | ***Y*** | ***Z*** |
| R Anterior insula | 42 | 12 | -5 |
| L Anterior insula | -40 | 9 | 1 |
| R Thalamus | 10 | -11 | 0 |
| L Thalamus | -10 | -14 | 0 |
| R Parietal operculum | 54 | -27 | 21 |
| L Parietal operculum | -55 | -25 | 19 |
| R Putamen | 24 | 9 | 6 |
| L Putamen | -24 | 14 | 3 |
| R Anterior cingulate | -2 | 33 | 24 |
| L Anterior cingulate | 6 | 33 | 16 |
| R Supplementary motor cortices | 53 | -1 | 46 |
| L Supplementary motor cortices | -37 | -10 | 43 |
| R Dorsolateral prefrontal cortex (dlPFC) | 40 | 34 | 40 |
| L Dorsolateral prefrontal cortex (dlPFC) | -40 | 34 | 40 |
| R Primary motor cortex M1 (precentral gyrus) | 38 | -18 | 45 |
| L Primary motor cortex M1 (precentral gyrus) | -36 | -19 | 48 |
| R Primary somatosensory cortex (S1)  (postcentral gyrus) | 42 | -28 | 57 |
| L Primary somatosensory cortex (S1)  (postcentral gyrus) | -31 | -40 | 66 |
| R Orbitofrontal gyrus (ventromedial pre-  frontal cortex, vmPFC) | 9 | 56 | -12 |
| L Orbitofrontal gyrus (ventromedial pre-  frontal cortex, vmPFC) | -11 | 38 | -19 |
| R Nucleus accumbens (NAc) | 15 | 8 | -9 |
| L Nucleus accumbens (NAc) | -17 | 3 | -9 |
| R Hippocampus | 28 | -22 | 14 |
| L Hippocampus | -29 | -19 | -15 |
| R Amygdala | 19 | -3 | -25 |
| L Amygdala | -15 | -4 | -22 |

R, right; L, left.
