# Supplementary material for: Increased functional connectivity between limbic brain areas in healthy individuals with high versus low sensitivity to cold pain: A resting state fMRI study
Source: PLoS One. 2022 Apr 20;17(4):e0267170. doi: 10.1371/journal.pone.0267170 (PMC9020745; doi:10.1371/journal.pone.0267170)
Supplement: S2 Table — (DOCX) [file pone.0267170.s002.docx]

**Table S2. Pain parameters in HSP and LSP in the entire study population (N=156).**

| **Pain tests** | **HSP**  **n=80** | **LSP**  **n=76** | ***T*** | ***P value*** | **Cohen’s *d*** |
| --- | --- | --- | --- | --- | --- |
| Cold pain threshold (CPT sec) | 3.8 ± 2.3 | 17.9 ± 11.6 | 10.6 | **<.001** | 1.6 |
| Cold pain intensity (NPS 0-100 at 1ºC) | 74.3 ± 18 | 35.8 ± 17 | -13.7 | **<.001** | 2.1 |
| Cold pain tolerance (sec) | 13.2 ± 4.7 | 180 ± 0.0 | 304 | **<.001** | - |
| Heat pain threshold (ºC) | 41.1 ± 3.5 | 45.1 ± 3.7 | 6.8 | **<.001** | 1.1 |
| Heat pain intensity (NPS 0-100 at 46.5 ºC) | 87.6 ± 19 | 49.7 ± 33.4 | -8.8 | **<.001** | 1.4 |
| Mechanical pain threshold (KPa) | 219.3 ± 110.6 | 426.9 ± 287.3 | 6.0 | **<.001** | 0.9 |
| Mechanical pain tolerance (KPa) | 542.8 ± 235.2 | 1216.4 ± 513.5 | 10.6 | **<.001** | 1.6 |

HSP, high sensitivity to pain; LSP, low sensitivity to pain; CPT, cold pressor test; TSA, thermal sensory analyser; NPS, numerical pain scale; KPa, kilopascal. Results are presented as mean ± standard deviation.
